# Moderate Modulation of Cardiac PGC-1α Expression Partially Affects Age-Associated Transcriptional Remodeling of the Heart

**DOI:** 10.3389/fphys.2018.00242

**Published:** 2018-03-21

**Authors:** Natasha Whitehead, Jonathan F. Gill, Marijke Brink, Christoph Handschin

**Affiliations:** ^1^Biozentrum, University of Basel, Basel, Switzerland; ^2^Department of Biomedicine, University of Basel and University Hospital Basel, Basel, Switzerland

**Keywords:** PGC-1α, aging, myocardium, remodeling, transcriptional regulation, heart

## Abstract

Aging is associated with a decline in cardiac function due to a decreased myocardial reserve. This adverse cardiac remodeling comprises of a variety of changes, including a reduction in mitochondrial function and a decline in the expression of the peroxisome proliferator-activated receptor γ coactivator 1α (PGC-1α), a central regulator of mitochondrial biogenesis and metabolic adaptation in the myocardium. To study the etiological involvement of PGC-1α in cardiac aging, we used mouse models mimicking the modest down- and upregulation of this coactivator in the old and the exercised heart, respectively. Young mice with reduced cardiac expression of PGC-1α recapitulated part of the age-related impairment in mitochondrial gene expression, but otherwise did not aggravate the aging process. Inversely however, moderate overexpression of PGC-1α counteracts numerous key age-related remodeling changes, e.g., by improving blood pressure, age-associated apoptosis, and collagen accumulation, as well as in the expression of many, but not all cardiac genes involved in mitochondrial biogenesis, dynamics, metabolism, calcium handling and contractility. Thus, while the reduction of PGC-1α in the heart is insufficient to cause an aging phenotype, moderate overexpression reduces pathological remodeling of older hearts and could thereby contribute to the beneficial effects of exercise on cardiac function in aging.

## Introduction

Aging causes a decline in cardiac function as a result of decreased myocardial reserve and adverse remodeling (Dai et al., 2016). Key molecular phenotypes of cardiac aging include alterations in stress response pathways (Lakatta, 1993), mitochondrial function (Judge et al., 2005; Dai et al., 2012a; Tocchi et al., 2015), cardiac energy metabolism (Lee et al., 2002; Lopaschuk et al., 2010), calcium signaling (Koban et al., 1998; Hobai and O'Rourke, 2001; Bers, 2006), contractility (Zile and Brutsaert, 2002; Strait and Lakatta, 2012), cardiomyocyte death (Kwak, 2013b) and extracellular matrix (ECM) remodeling (Nadal-Ginard et al., 2003; Kwak, 2013a; Horn and Trafford, 2016). These changes are mediated by a complex interconnected network of transcriptional and posttranslational processes that initially may be beneficial, aimed at the maintenance of cardiac function, but in the long term often are detrimental to the heart (Volkova et al., 2005), resulting in age-related cardiac remodeling with impaired cardiac reserve and thus increasing the risk for heart failure and other cardiovascular events.

Intriguingly, various heart pathologies have been associated with mitochondrial dysfunction and altered cardiac metabolism (Dillon et al., 2012). Thus, not surprisingly, mitochondrial gene expression is prominently altered in the aging myocardium (Anderson and Prolla, 2009). As a consequence, aged hearts often reveal a mitochondrial impairment (Ventura-Clapier et al., 2008; Barton et al., 2016) while adequate mitochondrial function and integrity is vital for cellular homeostasis and cardiac performance (Chaudhary et al., 2011). Unfortunately, the underlying molecular mechanisms of these and other age-related changes are largely unknown. Notably however, the aged heart shows reduced levels of the peroxisome proliferator-activated receptor y coactivator 1α (PGC-1α) as well as downstream transcription factor binding partners including the estrogen-related receptor α (ERRα), the peroxisome proliferator-activated receptor α (PPARα) and the mitochondrial transcription factor A (TFAM) (Finck and Kelly, 2006; Scarpulla, 2008; Vina et al., 2009; Dillon et al., 2012; Vega and Kelly, 2017).

The transcriptional network that is controlled by PGC-1α and its transcription factor partners is intrinsically linked to the control of mitochondrial biogenesis and metabolic adaptation in various tissues, including skeletal, and cardiac muscle (Dillon et al., 2012; Kupr and Handschin, 2015; Schnyder et al., 2017; Vega and Kelly, 2017). Loss-of-function animal models for heart PGC-1α exhibit an inability to meet the energy demands precipitated by increased cardiac work load, primarily due to reduced mitochondrial fatty acid oxidation and ATP synthesis efficiency (Arany et al., 2005; Leone et al., 2005; Lai et al., 2008; Lehman et al., 2008; Martin et al., 2014). An analogous reduction in PGC-1α expression has been reported in different cardiac pathologies, linked to a switch from oxidative metabolism to glycolysis (Finck and Kelly, 2006; Vega and Kelly, 2017). Conversely, high-level cardiac-specific overexpression also leads to impaired heart function, in this case accompanied by uncontrolled mitochondrial biogenesis, loss of sarcomere structure and a dilated cardiomyopathy (Lehman et al., 2000). Thus, deregulation of PGC-1α in either direction evokes adverse effects in the heart, demonstrating the critical role of this transcriptional coactivator in the maintenance of cardiac health (Anderson and Prolla, 2009). Importantly, a moderate increase in PGC-1α expression, e.g., as observed in some endurance exercise studies (O'Neill et al., 2007; Kim et al., 2008; Riehle et al., 2014; Vettor et al., 2014; Tam et al., 2015), or even the prevention of PGC-1α deterioration, could elicit beneficial effects in the heart. For example, low level overexpression of PGC-1α in the myocardium promotes an excitation-contraction (E-C) coupling phenotype that is prototypic for physiological hypertrophy of the heart, in addition to altering the expression of genes involved in the regulation of the circadian clock, heat shock, excitability, calcium signaling and contraction (Mutikainen et al., 2016).

In this study, we used both gain- and loss-of-function models for moderate overexpression and reduction of PGC-1α in the heart, respectively, to study the involvement of this coactivator in controlling cardiac aging. While a modest downregulation of PGC-1α in young animals was linked to a mitochondrial gene expression signature similar to that observed in an old heart, this reduction was insufficient to elicit a premature aging phenotype in regards to other aspects of age-related cardiac remodeling. Inversely however, a moderate elevation of PGC-1α blunted or prevented a broad range of age-associated changes in the heart of old mice.

## Materials and methods

### Experimental animals

Male, 3 month old and 24 month old PGC-1α muscle-specific knockout (MKO) and transgenic (MTg) mice of the same C57BL/6 background as well as their respective littermate controls (WT) were obtained from in-house breeding of previously described lines. The reduction of PGC-1α gene expression in the MKO animals resulted from human skeletal actin (HSA) promoter-driven cre expression in floxed PGC-1α mice (Perez-Schindler et al., 2013). The overexpression of PGC-1α in the MTg mice resulted from the muscle creatine kinase (MCK) promoter to drive the expression of a PGC-1α transgene (Lin et al., 2002). Both animal models depict their primary change in skeletal muscle, but due to the low expression of the HSA and MCK promoters in cardiomyocytes, a more modest reduction and elevation, respectively, of PGC-1α in the heart is achieved. The MKO mice were homozygous for the floxed PGC-1α and heterozygous for the HSA-cre allele. The MTg animals were heterozygous for the MCK-PGC-1α transgene. All mice were kept in a conventional facility under a 12/12 h light/dark cycle and had free access to food and water. This study was carried out in accordance with the principles of the Basel Declaration and with Federal and Cantonal Laws regulating the care and use of experimental animals in Switzerland, as well as institutional guidelines of the Biozentrum and the University of Basel. The protocol with all methods described here was approved by the “Kantonales Veterinäramt” of the Kanton Basel-Stadt, under consideration of the well-being of the animals and the 3R principle.

### Blood pressure measurements

Arterial blood pressure (BP) was measured with a non-invasive tail cuff system (BP-2000 blood pressure analysis system, Visitech Systems). Blood pressure was measured over 5 consecutive days with 5 pre-measurements and 15 measurements per day. Mice were rotated in terms of their position and measurements were performed at the same time each day.

### Tissue collection

Animals were sacrificed by carbon dioxide inhalation and mouse length was measured from the tail base to the nose with mice lying straight on the dissection plate. Immediately after death, hearts were dissected, washed in Dulbecco's phosphate buffered saline (PBS, Sigma-Aldrich) and cut transversely in half. The upper half was flash frozen in liquid nitrogen for RNA and protein extraction. The lower part was frozen in cooled isopentane and embedded in 7% Tragacanth for preparation of cryosections. Transverse heart area and collagen quantification were assessed by consecutive cutting of the sections starting at the tip until the left ventricle was reached. Transverse heart area was determined on 5–6 sections per heart in at least 4 mice per group by using a threshold in which the entire tissue could be visualized after conversion of the color into primary images and using the functions “Analyze” and “Measure” to determine area and perimeter in Fiji.

### RNA isolation and gene expression analysis

Total cardiac mRNA was extracted from frozen, crushed samples with the help of Lysing matrix tubes (MP Biomedicals), 1 ml Tri reagent (Sigma-Aldrich) and FastPrep FP120. The procedure was carried out according to the manufacturer's instructions. RNA concentration and purity was measured with a NanoDrop ND-1000 Spectrophotometer (Thermo Scientific). The RNA purity was determined with the ratio of 260/280 nm and 260/230 nm. A purification step was carried out on samples with purities beneath 1.7.

RNA quality was assessed using a 2100 Bioanalyzer (G2938B, Agilent Technologies). The lowest RIN detected was 8.2. 400 ng of total RNA was treated with DNase I (Invitrogen) and then reverse transcribed into cDNA using SuperScript II Reverse Transcriptase (Invitrogen) and random hexanucleotide mix (Roche).

The relative expression levels of each gene of interest were quantified with the ΔΔCT method using FastStart Essential DNA Green Master (Roche) and the LightCycler 480 system (Roche). The average of the values obtained for the housekeeping genes TATA-binding protein (TBP), hypoxanthine guanine phosphoribosyl transferase (HPRT) and 18S rRNA was used for normalization Primer sequences are listed in Table 1.

**Table 1:** Primer pairs for real-time RT-qPCR analysis.

**Gene target**	**Name**	**Forward primer sequence (5′ - 3′)**
		**Reverse primer sequence (5′ - 3′)**
18S	18S ribisomal RNA	AGTCCCTGCCCTTTGTACACA
		CGATCCGAGGGCCTCACTA
ACTC1	Actin, Alpha, Cardiac Muscle 1	CTGGATTCTGGCGATGGTGTA
		CGGACAATTTCACGTTCAGCA
ATP5b	ATP Synthase, H+ Transporting, Mitochondrial F1 Complex, Beta Polypeptide	ACGTCCAGTTCGATGAGGGAT
		TTTCTGGCCTCTAACCAAGCC
CKMT2	Creatine Kinase, Mitochondrial 2	CCACACCAGGGTGATCTCAAT
		TCGAGGGGCAAGTCAAAATGT
COL I	Collagen Type I Alpha 1 Chain	CTTCACCTACAGCACCCTTGT T
		TGACTGTCTTGCCCCAAGTTC
COL III	Collagen Type III Alpha 1 Chain	CACCCTTCTTCATCCCACTCT T
		TGCATCCCAATTCATCTACGTT
CPT1β	Carnitine Palmitoyltransferase 1B	ATCATGTATCGCCGCAAACT
		CCATCTGGTAGGAGCACATGG
CS	Citrate synthase	CCCAGGATACGGTCATGCA
		GCAAACTCTGCGTGACAGGAA
DLP1	Dynamin like protein-1	GCGCTGATCCCGCGTCAT
		CCGCACCCACTGTGTTGA
ERRα	Estrogen Related Receptor Alpha	CGGTGTGGCATCCTGTGA
		CTCCCCTGGATGGTCCTCTT
FIS1	Fission, Mitochondrial 1	GCCCCTGCTACTGGACCAT
		CCCTGAAAGCCTCACACTAAGG
PYGM	Glycogen phosphorylase	CTTAGCCGGAGTGGAAAATGT
		GTAATCCTCCGGAGTAGCCAA
HPRT	Hypoxanthine Phosphoribosyltransferase 1	ATGCCGAGGATTTGGAAAAAGTG
		TGACATCTCGAGCAAGTCTTTCA
LCAD	Long Chain Acyl-CoA Dehydrogenase	CCAGCTAATGCCTTACTTGGAGA
		GCAATTAAGAGCCTTTCCTGTGG
MCAD	Medium Chain Acyl-CoA Dehydrogenase	AACACTTACTATGCCTCGATTGCA
		CCATAGCCTCCGAAAATCTGAA
MFN1	Mitofusin 1	CTGCTTCCTGAGTGTCGAGG
		GCATGGGCCAGCTGATTAAC
MFN2	Mitofusin 2	GGTCAGGGGTATCAGCGAAG
		TTGTCCCAGAGCATGGCATT
mTOR	Mammalian Target Of Rapamycin	TGATGTGCCGAGACCTTGAG
		GCTTGGATGTGATGACTTGCA
NCX1	Sodium-calcium exchanger 1	GGTGAACTGCCTCCAGAGAG
		GTGCCAGACACCGTATCCTT
OPA1	OPA1, Mitochondrial Dynamin Like GTPase	CTTGCCAGTTTAGCTCCCGA
		CAATTTGGGACCTGCAGTGAA
OPA2	Optic Atrophy 2 (Obscure)	CCCAGCTCAGAAGACCTTGC
		CCAGGTGAACCTGCAGTGAA
PGC-1α Ex3-5	Peroxisome proliferator-activated receptor gamma coactivator 1-alpha	AGCCGTGACCACTGACAACGAG
		GCTGCATGGTTCTGAGTGCTAAG
PGC-1β	Peroxisome proliferator-activated receptor gamma coactivator 1-beta	CCATGCTGTTGATGTTCCAC
		GACGACTGACAGCACTTGGA
PLN	Phospholamban	ATGACGACGATTCAAATCTCTTGG
		TGGGTTTGCAAAGTTAGGCATAA
PPARα	Peroxisome Proliferator Activated Receptor Alpha	GCGTACGGCAATGGCTTTAT
		ACAGAACGGCTTCCTCAGGTT
SERCA2	ATPase Sarcoplasmic/Endoplasmic Reticulum Ca^2+^ Transporting 2	TCAGCAGGAACTTTGTCACC
		GGGCAAAGTGTATCGACAGG
SOD2	Superoxide Dismutase 2, Mitochondrial	TGAACAATCTCAACGCCACCGAG
		TGAACTTCAGTGCAGGCTGAAGAG
TBP	TATA-Box Binding Protein	TGCTGTTGGTGATTGTTGGT
		CTGGCTTGTGTGGGAAAGAT
TERF1	Telomeric repeat binding factor 1	CATGGACTACACAGACTTAC
		ATCTGGCCTATCCTTAGACG
TERF2	Telomeric repeat binding factor 2	AGCTGATTCCAAGGGTGTGA
		GGTTATGCAGTGTCTGTCGC
TFAM	Transcription factor A, mitochondrial	GGTCGCATCCCCTCGTCTA
		GGATAGCTACCCATGCTGGAAA
TNNI3	Toponin I3, cardiac type	TCTGCCAACTACCGAGCCTAT
		CTCTTCTGCCTCTCGTTCCAT
VEGF	Vascular endothelial grwoth factor	CTGTGCAGGCTGCTGTAACG
		GTTCCCGAAACCCTGAGGAG
α-MHC	Myosin heavy chain alpha isoform	CTACGCGGCCTGGATGAT
		GCCACTTGTAGGGGTTGAC
β-MHC	Myosin heavy chain beta isoform	TTGAGAATCCAAGGCTCAGC
		CTTCTCAGACTTCCGCAGGA

### Protein isolation and western blotting

Cardiac proteins were extracted from 30 mg of crushed samples using 300 μl ice-cold tissue lysis buffer [50 mM Tris-HCl (pH 7.5), 1 mM EDTA, 0.5 mM EGTA, 1% NP-40 substitute, 150 mM NaCl, 0.2% Na-deoxycholate, 1 mM dithiothreitol (DTT), complete mini protease inhibitor (Roche), PhosStop easy Pack phosphatase inhibitor (Roche), 10 mM nicotinamide], Pellet Pestel Motor (Kontes) and MISONIX ultrasonic liquid processor. Then, the samples were shaken for 30 min at 1,300 rpm at 4°C and centrifuged at 13,000 g for 10 min at 4°C. The protein concentration of the supernatant was determined by the albumin standard method (Thermo Scientific), samples were diluted in lysis buffer to a final concentration of 3 μg/μl containing 1x Laemmli Sample Buffer (Bio-Rad) and 20% β-mercaptoethanol (Bio-Rad) and subsequently boiled for 5 min.

Thirty microgram of proteins were loaded and separated on Mini-PROTEAN TGX Stain-free Precast gels (4–20%, Bio-Rad). The transfer was carried out for 1 h at 100 V onto nitrocellulose membranes. Membranes were blocked for 1 h in either 5% bovine albumin serum (BSA) or in 5% BSA with Tris buffered saline and Tween 20 (TBST) for 60 min and incubated overnight at 4°C with primary antibodies (see Table 2 for details): Myosin (skeletal, slow) (200 kDa; Sigma-Aldrich) (diluted 1:5,000) and Caspase 3 (17,19,35 kDa; Cell Signaling) (diluted 1: 1,000), both in 3% BSA. For protein detection, membranes were incubated for 1 h with the secondary antibody: Anti-Mouse (Dako) diluted 1:10,000 in 3% BSA, respectively 3% milk. Antibody detection was carried out using an appropriate chemiluminescence horseradish peroxidase (HRP) substrate detection kit (Thermo Scientific). The imaging and quantification of Western blots was done with the Fusion software (Fusion). Total protein was used as a loading control. Imaging of total protein was carried out on gel and membrane using a stain-free enabled imaging system (Fusion). Representative Western blots of two animals per group are shown, quantification was performed on *n* = 6 per group.

**Table 2:** Antibody conditions.

	**Blocking solution**	**Conditions for primary antibody**	**Conditions for secondary antibody**
Myosin (skeletal, slow) (200 kDa; Sigma-Aldrich)	5% BSA	Ab diluted 1:5,000 in 3% BSA and 0.2% Na-azide	Anti-Mouse (Dako) diluted 1:10,000 in 3 % BSA
Caspase 3 (17–19, 35 kDa; Cell Signaling)	5% milk	Ab diluted 1:1,000 in 3% BSA and 0.2% Na-azide	Anti-Rabit (Dako) diluted 1:10,000 in 3 % milk

### Picrosirius red staining

Eight micro meters-thick heart cryo-sections were thawed and stained for 1 h in Picrosirius Red (0.5 g Sirius Red under the name Direct Red 80, Sigma-Aldrich, in 500 ml 1.3% saturated aqueous solution of picric acid, Sigma-Aldrich). Sections were washed in acidified water (5 ml glacial acetic acid in 1 l distilled water), dehydrated by 3 incubations with 100% ethanol, cleared in xylene and mounted with Histomount (Invitrogen). Quantification of the collagen amount from the Picrosirius Red staining was carried out with the software Ilastik 1.1 and Fiji. Briefly, 12 images representing the true staining (collagen = red; background = yellow) as well as artifacts such as dirt, wrinkles, blood, image noise, blood vessels, unevenness of staining were cropped to train the program illastik to recognize these problem zones and create a masked image allowing an equal quantification of collagen of all used images (5–6 whole sections per group, sections obtained from different animals). The area covered by collagen in these masked images was measured using the software Fiji.

### Hydroxyproline assay

Ten microgram of frozen heart tissue was diluted in 100 μl nuclease free water and homogenized using the MISONIX ultrasonic liquid processor and transferred to a pressure-safe pyrex glass tube. The same amount of 37% HCl was added to the samples, which were then heated at 120°C for 3 h. For each assay, 10 μl of sample was transferred to a 96-well plate and evaporated at 60°C to dryness. Chloramine T (55 mM chloramine T), 10% 2-propanol in acetate citrate buffer (0.8M sodium acetate trihydrate, 240 mM citric acid, 1.2% glacial acetic acid, 850 mM NaOH in 1 l ddH_2_O) and Ehrlich's reagent [10 mM p-dimethylaminobenzaldehyde in 2-propanol/perchloric acid (2:1 v/v)] were added and the absorbance at 560 nm was measured with Infinite M1000 (Tecan). The amount of hydroxyproline was calculated with the help of a hydroxyproline standard (Sigma-Aldrich).

### Statistical analysis

All statistical calculations were done with GraphPad PRISM version 6.07. Statistical significance was calculated using 2-Way ANOVA and all values were displayed as means ± standard errors of the means (SEM). ^*^/#: *p* < 0.05; ^**^/##: *p* < 0.01; ^***^/###: *p* < 0.001; ^****^/####: *p* < 0.0001 indicates age/genotype related significant differences. In the case of p values close, but slightly above to the significance cut-off of *p* < 0.05, actual *p*-values are indicated.

## Results

### Moderate overexpression of PGC-1α prevents age-related increases in diastolic blood pressure

**Figure 1:**
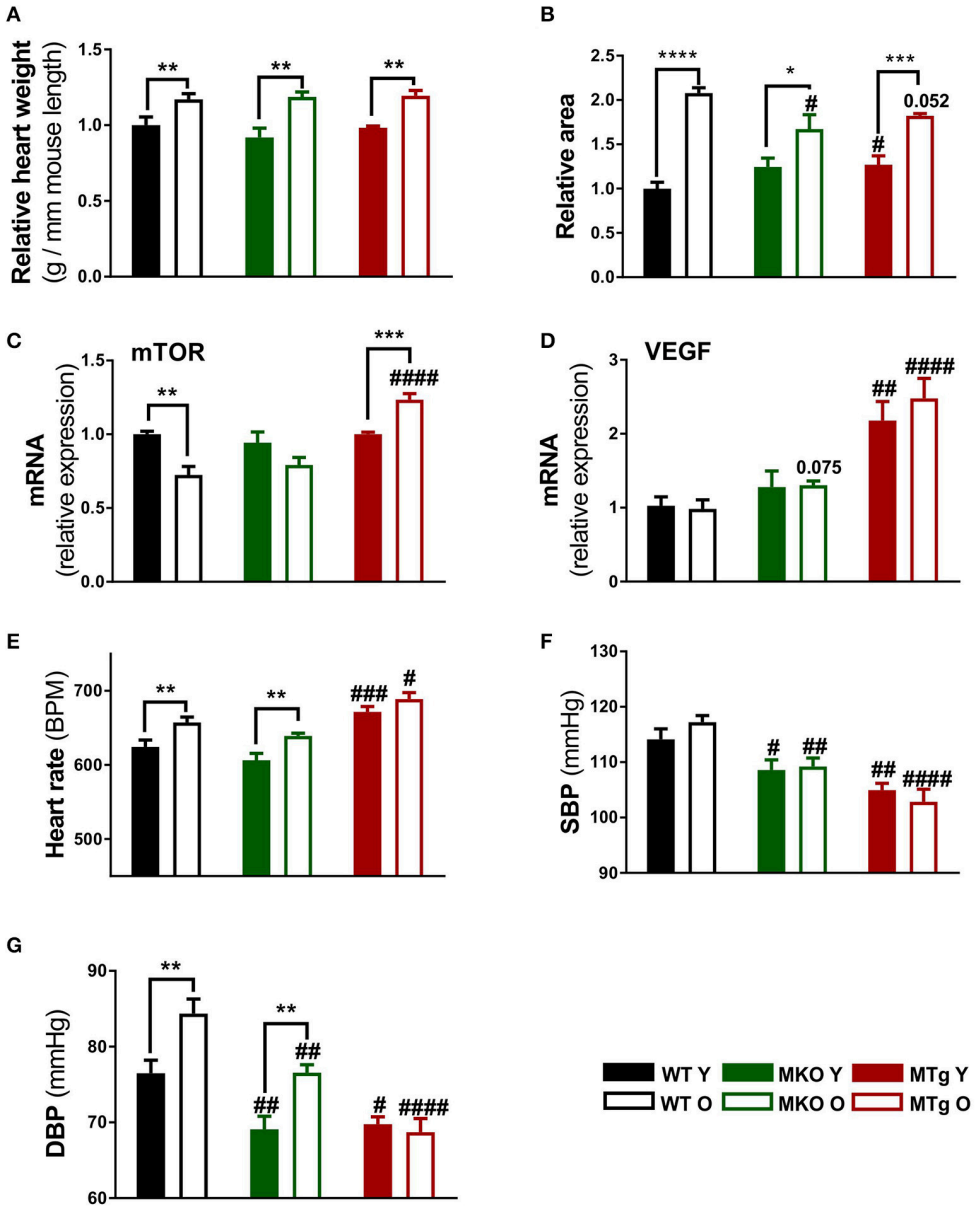
Moderate PGC-1α modulation reduces blood pressure. **(A)** Heart weight to mouse length ratios were assessed after sacrifice at 3 and 24 months of age for the young (filled bars) and old (open bars) mice, respectively (*n* = 6/group). **(B)** Transverse heart area was measured on microscopy sections using Fiji software (*n* = 4/group). Relative area refers to normalization to the area measured in WT Y animals. **(C,D)** Gene expression of mTOR and VEGF (*n* = 6/group). **(E–G)** Heart rate, systolic and diastolic blood pressure were measured with BP-2000 Blood Pressure Analysis System (Visitech Systems) (*n* = 10–12/group). Data are means ± SEM. Significant differences (*p* < 0.05) associated with age and phenotype are indicated by asterisks (^*^) and by hashtags (#, compared to WT), respectively (^*^/#: *p* < 0.05; ^**^/##: *p* < 0.01; ^***^/###: *p* < 0.001; ^****^/####: *p* < 0.0001 indicates age/genotype related significant differences). DBP, diastolic blood pressure; mTOR, mammalian target of rapamycin; O, old; SBP, systolic blood pressure; Y, young; WT, wildtype; MKO, muscle knockout; MTg, muscle transgenic.

First, general cardiovascular traits were analyzed in our models of moderate PGC-1α deficiency and overexpression in comparison to WT mice. All three mouse lines exhibited an age-linked increase in heart weight (Figure 1A) associated with an elevated cardiac area (Figure 1B). Intriguingly, the cardiac area was already significantly larger in the young PGC-1α MTg mice while the old MKO animals showed a reduced cardiac area compared with the respective age-matched WT mice (Figure 1B). Despite the parallel trend for heart weight and area, the age-related decrease in gene expression of the growth- and hypertrophy-regulating kinase mammalian target of rapamycin (mTOR) in the WT group was diametrically opposite in the MTg mice (Figure 1C). Furthermore, a strong increase in vascular endothelial growth factor (VEGF) gene expression was observed in both young and old MTg mice (Figure 1D), which might be indicative of improved tissue vascularization in this model. Even though some studies reported depressed heart rates with increased age (Chaudhary et al., 2011; Moghtadaei et al., 2016), we observed an increase in the WT as well as MKO animals (Figure 1E). The MTg mice exhibited an increased heart rate already at young age (Figure 1D). Overall, blood pressures were lower in MKO and MTg mice than in WT mice (Figures 1F,G). Systolic blood pressures did not change with age, whereas as published, diastolic pressures were significantly higher in the old than in the young mice of (Figure 1F). Notably, there is an age-dependent increase of diastolic blood pressure in the WT and MKO lines, which is prevented by the moderate PGC-1α overexpression in the MTg mice (see Gill et al., 2018, replicated in Figure 1G).

### Moderate overexpression of PGC-1α counteracts age-associated ECM remodeling

**Figure 2:**
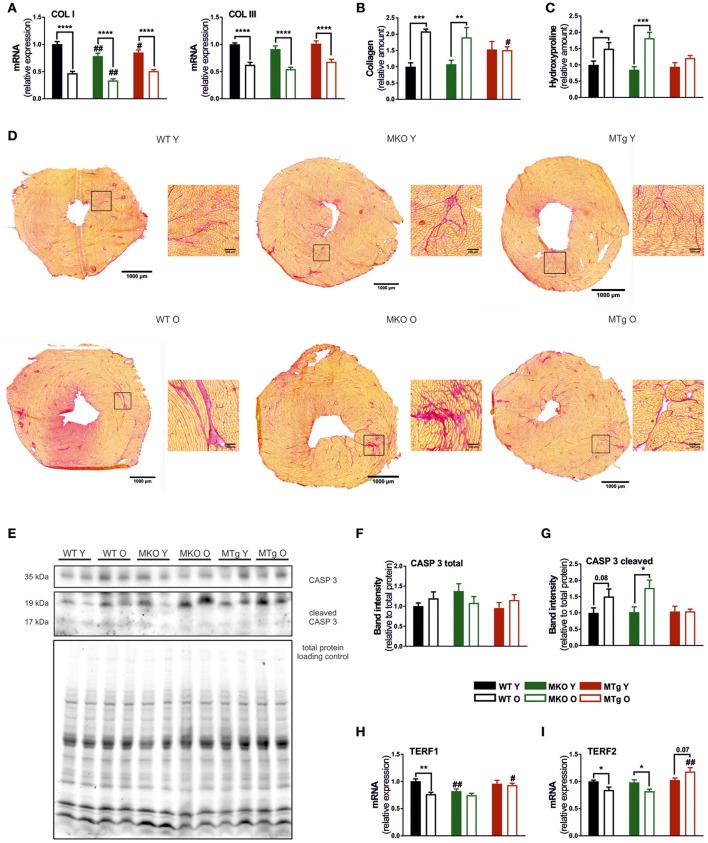
PGC-1α overexpression reduces age-dependent collagen accumulation and the age-induced expression of telomere-modulating genes. **(A)** Gene expression of collagen type I and III (*n* = 6/group). **(B)** Relative collagen content measured with Picrosirius Red staining (*n* = 5–6/group). **(C)** Relative content of hydroxyproline measured with hydroxyproline assay (*n* = 6/group). **(D)** Picrosirius Red staining done on 8 μm thick cryosections, red indicates collagen. **(E)** Western blot of caspase 3 and cl. caspase 3, normalized to total protein loading control. Western blot quantification (*n* = 6/group) of **(F)** caspase 3 and **(G)** cleaved caspase 3 (*n* = 6/group). **(H,I)** Expression of TERF1 and TERF2 mRNA (*n* = 6/group). Data are means ± SEM of values from each group. Significant age/genotype-associated differences (*p* < 0.05) are indicated by asterisks (^*^) and by hashtags (#, compared to WT) respectively (^*^/#: *p* < 0.05; ^**^/##: *p* < 0.01; ^***^p < 0.001; ^****^p < 0.0001 indicates age/genotype related significant differences). CASP, caspase; COL, collagen; O, old; TERF, telomeric repeat-binding factor; Y, young.

In older hearts, programmed cell death and necrosis induce not only loss of contractile tissue, but also a reactive compensatory hypertrophy of remaining cardiomyocytes, accumulation of collagen and fibrosis (Jugdutt, 2003). In all three mouse lines, mRNA expression of collagen I and III was reduced with age (Figure 2A). Strikingly however, picrosirius red staining revealed that an accumulation of collagen protein in aged WT mice was completely abrogated in the MTg animals (Figures 2B–D). This discrepancy between transcript and protein levels could stem from an adaptive, repressive process to reduce collagen expression, which however is dominated by the altered balance between ECM synthesis and degradation due to aging-induced modulation of matrix metalloprotease (MMP) and tissue inhibitors of metalloproteinases (TIMP) activities (Kwak, 2013a). Apoptosis may be an additional potential initiator of ECM remodeling. In line with the effect on collagen accumulation, moderate overexpression of PGC-1α in the MTg model prevented the age-linked increase in cleaved caspase 3, a known marker of apoptosis (Figures 2E–G). Finally, moderate elevation of PGC-1α also counteracted the age-associated diminished gene expression of the telomeric repeat-binding factor 1 and 2 (TERF1 and TERF2) (Figures 2H,I), two proteins that protect mammalian telomers (Palm and de Lange, 2008; Bernardes de Jesus and Blasco, 2012; Moslehi et al., 2012). The young MKO mice already exhibit levels of TERF1 corresponding to old WT animals (Figure 2H).

### Moderate PGC-1α overexpression counteracts the age-associated downregulation of mitochondrial gene expression

**Figure 3:**
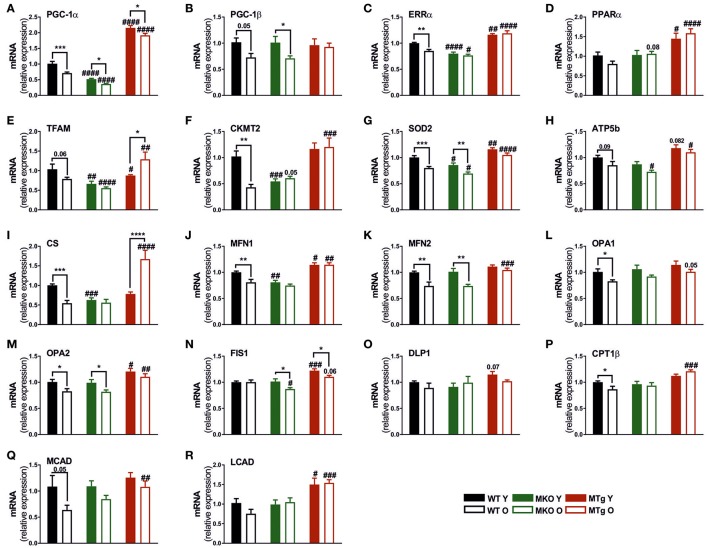
PGC-1α overexpression protects against the age-related reduction of genes involved in mitochondrial biogenesis, dynamics, oxidative phosphorylation and fatty acid oxidation**. (A–F)** Gene expression profile of genes involved in mitochondrial biogenesis and function (*n* = 6/group). **(G–I)** Gene expression levels of mitochondrial superoxide dismutase (SOD2), OXPHOS and TCA cycle genes (*n* = 6/group). **(J–M)** Mitochondrial fusion gene expression profile (*n* = 6/group). **(N,O)** Mitochondrial fission gene expression profile (*n* = 6/group). **(P–R)** Fatty acid β-oxidation (FAO) gene expression profile. Data are means ± SEM of values from each group. Significant age/genotype-associated differences (*p* < 0.05) are indicated by asterisks (^*^) and by hashtags (#, compared to WT) respectively (^*^/#: *p* < 0.05; ^**^/##: *p* < 0.01; ^***^/###: *p* < 0.001; ^****^/####: *p* < 0.0001 indicates age/genotype related significant differences). ATP5B ATP synthase, H+ transporting, mitochondrial F1 complex, beta polypeptide; CKMT2, creatine kinase, mitochondrial 2; CPT1β, carnitine palmitoyltransferase 1β; CS, citrate synthase; DLP1, dynamin-like protein 1; FIS1, fission, mitochondrial 1; LCAD, long chain acyl-CoA dehydrogenase; MCAD, medium chain acyl-CoA dehydrogenase; MFN, mitofusin; O, old; Opa, mitochondrial dynamin like GTPase; SOD2, mitochondrial superoxide dismutase; TCA, tricaboxylic acid; Y, young.

Similar to other heart pathologies, cardiac aging leads to the decreased expression of genes involved in mitochondrial biogenesis and function (Dai et al., 2012b). In line, the level of several mitochondrial transcripts was reduced in old compared to young WT mice (Figure 3), notably also the levels of PGC-1α and the estrogen-related receptor α (ERRα), one of the major transcription factor binding partners of PGC-1α in the regulation of metabolic gene expression (Huss et al., 2002). This reduction was also observed for some of these genes in the MKO mice (Figure 3) while importantly, the transcript levels of several mitochondrial genes were already reduced in young MKO animals comparable to old WT expression (Figure 3). Moreover, mitochondrial gene expression was further reduced in MKO animals compared to old WT mice. Strikingly, moderate overexpression of PGC-1α not only almost completely blunted the age-associated reduction in gene expression, but was also sufficient to raise the transcript levels of most genes in young and old MTg hearts above those of young WT mice (Figure 3). These patterns were not only observed in genes encoding proteins important for mitochondrial biogenesis, Krebs cycle and oxidative phosphorylation (OXPHOS) (Figures 3A–I), but also in mitochondrial fission and fusion gene expression (Figures 3J–O). WT mice showed an age-dependent decrease in the fatty acid β-oxidation (FAO) transcripts (Figures 3P–R) indicating a possible decreased reliance of cardiomyocytes on lipid metabolism in line with the reduction in OXPHOS gene expression. Interestingly, the FAO gene expression did not follow the WT pattern in the MKO mice. More consistently, we observed a pattern of increased transcript levels of the FAO genes, and an abrogation of the age effect in the MTg mice compared to the age-matched WT animals (Figures 3P–R).

### Moderate PGC-1α overexpression induces a gene expression profile favoring calcium handling and contractile function

**Figure 4:**
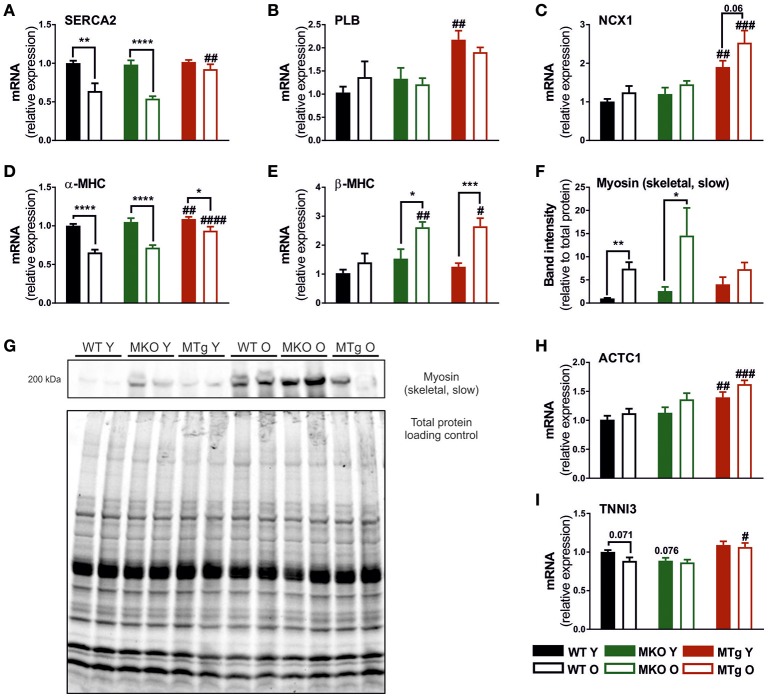
Increased PGC-1α blunts the age-associated regulation of calcium homeostasis and contractile genes. Gene expression levels of **(A)** SERCA2, **(B)** PLN, **(C)** NCX1, **(D)** α-MHC, **(E)** β-MHC, **(H)** ACTC1, and **(I)** TNNI3 (*n* = 6/group). **(F,G)** Western blot and quantification (*n* = 6/group) of myosin (skeletal, slow), which represents β-MHC in the heart, normalized to total protein loading control. Data are means ± SEM of values from each group. Significant age/genotype-associated differences (*p* < 0.05) are indicated by asterisks (^*^) and by hashtags (#, compared to WT) respectively (^*^/#: *p* < 0.05; ^**^/##: *p* < 0.01; ^***^/###: *p* < 0.001; ^****^/####: *p* < 0.0001 indicates age/genotype related significant differences). ACTC1, actin, alpha, cardiac muscle 1; PLB, phospholamban; MHC, myosin heavy chain; NCX1, sodium/calcium exchanger protein 1; O, old; SERCA2, sarcoplasmic/endoplasmic reticulum Ca^2+^ transporting ATPase 2; TNNI3, troponin I3, cardiac type; Y, young.

In addition to mitochondrial gene expression, age-related cardiac changes also include a decline in transcription of genes encoding proteins for calcium handling and contractility (Koban et al., 1998; Hobai and O'Rourke, 2001; Zile and Brutsaert, 2002; Bers, 2006; Strait and Lakatta, 2012). The age-related decrease in sarcoplasmic/endoplasmic reticulum Ca^2+^ transporting ATPase 2 (SERCA2) gene expression was observed in WT and MKO mice, but was completely prevented by the moderate overexpression of PGC-1α in the MTg mice (Figure 4A). Interestingly, the MTg animals also exhibited elevated transcript levels of phospholamban (PLB) and sodium/calcium exchanger protein 1 (NCX1), two additional genes involved in the regulation of sarcoplasmic reticulum (SR) calcium handling (Figures 4B,C), which could indicate overall improved calcium clearance. A blunting of the age effect in the MTg mice was also observed for α-myosin heavy chain (α-MHC) expression (Figure 4D). Curiously, an increase in β-MHC transcript levels was found in both old MKO and MTg, but not WT animals (Figure 4E). In contrast however, aging increased slow myosin protein levels only in WT and MKO mice, while the overexpression of PGC-1α in the MTg animals prevented this switch (Figures 4F,G). Finally cardiac α-actinin (ACTC1) expression was elevated in young and old MTg mice (Figure 4H), while cardiac troponin I3 (TNNI3) transcripts were significantly increased only in old MTg animals compared to the WT and MKO counterparts (Figure 4I).

## Discussion

Aging affects the function of every organ in our body. In the heart, age-related remodeling is closely linked to an increased risk for cardiac disease and heart failure. Similar to other pathological contexts, a shift in metabolism from oxidative substrate usage toward glycolysis, accompanied by a reduction in mitochondrial number and function, has been described in older hearts. This metabolic switch is often observed together with a decrease in the expression of PGC-1α (Dorn et al., 2015). Indeed, we have also observed an age-linked decline in PGC-1α expression (Figure 3A). While the mechanistic underpinnings of this regulation are unclear, it is conceivable that analogous to skeletal muscle, cardiac PGC-1α gene expression is based on the aging-associated reduction in contractile function, but maybe also due to changes in the neuroendocrine milieu, or metabolic properties. Inversely, exercise, which exerts beneficial effects on cardiac function, elevates PGC-1α gene expression in cardiac and skeletal muscle (Vega et al., 2017). We have now used transgenic models of moderate reduction and overexpression, respectively, to study the involvement of PGC-1α in the cardiac aging process. Notably, relatively small changes in PGC-1α gene expression resulted in striking changes in the transcriptional program in the young and old heart (summarized in Table 3). Determination of mitochondrial function would be of interest in future studies, since several aspects of mitochondrial respiration are altered in old skeletal muscle of gain- and loss-of-function models for PGC-1α (Gill et al., 2018). Strikingly, moderate overexpression of PGC-1α in the heart in the range of 1.5- to 2-fold quantitatively mimicking the increase in PGC-1α gene expression after exercise (O'Neill et al., 2007; Kim et al., 2008; Riehle et al., 2014; Vettor et al., 2014; Tam et al., 2015) blunted or prevented various aspects of age-associated transcriptional cardiac remodeling. As a key regulatory factor in the control of mitochondrial biogenesis and oxidative metabolism, PGC-1α overexpression resulted in a marked elevation of a number of mitochondrial and other metabolic genes. The modulation of several mitochondrial fission and fusion genes could indicate that PGC-1α is linked to higher mitochondrial dynamics and ultimately healthier mitochondria in cardiac myocytes (Chan, 2006; Chaudhary et al., 2011). Moreover, PGC-1α could reduce the levels of reactive oxygen species (ROS) (St-Pierre et al., 2006), which increase in the aged heart, cause oxidative damage to proteins, lipids and DNA, leading to the dysregulation of redox-sensitive signaling pathways (Judge et al., 2005). Such a PGC-1α-dependent effect has for example been described in diabetic nephropathy (Guo et al., 2015). A moderate elevation of PGC-1α in line with that observed in the trained heart thus seems sufficient to mitigate the aging-associated deterioration in mitochondrial function and cellular metabolism. Of note, superphysiological overexpression of cardiac PGC-1α has been linked to exacerbated mitochondrial biogenesis replacing myofibrillar structure, and ultimately leading to cardiomyopathy (Lehman et al., 2000; Russell et al., 2004).

**Table 3:** Overview of age-related changes in WT, MKO, and MTg groups and of the MKO and MTg compared to WT animals.

**Examined parameters**		**WT**	**MKO**			**MTg**		
		**Aging**	**Aging**	**Y**	**O**	**Aging**	**Y**	**O**
General physiology	Heart weight	↑	↑	nsd	nsd	↑	nsd	nsd
	Diastolic BP	↑	↑	−	−−	→	−−	−−−
ECM	Collagen (protein)	↑	↑	nsd	nsd	→	nsd	–
	Cl. caspase 3	↑	↑	nsd	nsd	→	nsd	nsd
Mitochondrial function	Mit. genes	↓	→	−−	−−	→	++	+++
	Mit. dynamics	↓	↓	nsd	nsd	→	+	+
Contractile function	SERCA2	↓	↓	nsd	nsd	→	nsd	++
	α-MHC	↓	↓	nsd	nsd	↓	++	++++
	β-MHC (protein)	↑	↑	nsd	nsd	→	nsd	nsd
Glucose metabolism	Glut1	→	↑	nsd	++++	→	nsd	++
	PFK	↑	→	nsd	−−−−	→	++++	++++
FA metabolism	CPT1β	↓	→	nsd	nsd	→	nsd	++
	LCAD	↓	→	nsd	nsd	→	+	++
	MCAD	(↓)	→	nsd	nsd	→	nsd	++

*Aging effects within the groups: 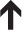,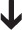: Aging effect seen as up-/downregulation of gene/ protein expression; 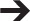: No aging effect. Moderate PGC-1α downregulation mainly follows the aging pattern seen in the WT animals whereas moderate PGC-1α upregulation counteracts many of these observed aging effects. (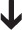) indicates a trend (p = 0.05)*.

*Gene expressions compared to age-matched WT: nsd, no significant difference; −/+: p < 0.05; −−/++: p < 0.01; −−−/+++: p < 0.001; −−−−/++++: p < 0.0001 down− (−) and upregulation (+), respectively*.

*BP, blood pressure; cl., cleaved; CPT1β, carnitine palmitoyltransferase 1β; FA, fatty acid; Glut, glucose transporter; LCAD, long chain acyl-CoA dehydrogenase; Mit., mitochondrial; MHC, myosin heavy chain; O, old; PFK, phosphofructokinase; SERCA2, sarcoplasmic/endoplasmic reticulum Ca^2+^-transporting ATPase 2; Y, young*.

Reduced mitochondrial function in aging has been associated with enhanced apoptosis, ECM remodeling and fibrosis (Chaudhary et al., 2011; Martin-Fernandez and Gredilla, 2016). The mitigation of age-associated cleavage of caspase 3, accumulation of collagens and expression of TERF2 in the MTg animals implies a broad effect of cardiac PGC-1α to reduce different aspects of this adverse remodeling. A similar outcome has been reported in a mouse model for pressure overload-induced cardiac hypertrophy, in which PGC-1α reduced apoptosis and fibrosis in severely stressed hearts (Pereira et al., 2014). Inversely, adequate mitochondrial function is essential to provide the primary source of energy that fuels the contractile apparatus according to the cellular demand (Chan, 2006). Moreover, tightly controlled calcium handling, and appropriate expression of contractile proteins are crucial for normal heart function. In the MTg mice, PGC-1α prevented the drop in SERCA2 expression, which is downregulated with age and other pathological cardiac conditions (Lompre et al., 1991). The concomitant increase in PLB and NCX1 transcript levels suggest a broader PGC-1α-dependent remodeling of cardiac calcium handling, analogous to the regulation of SR calcium homeostasis in skeletal muscle (Summermatter et al., 2012). This hypothesis is in line with previous findings describing that increased PGC-1α levels improve contractile and diastolic performance of cardiomyocytes by increasing calcium reuptake into the SR due to elevated SERCA2 activity, thereby preventing cellular calcium overload in the heart (Chen et al., 2010). In another study using the MTg animals as a model for modest cardiac elevation of PGC-1α, a strengthening of E-C coupling inducing favorable changes in excitability, calcium signaling and contraction has been reported (Mutikainen et al., 2016). Thus, together with the prevention of the aging-linked shift in contractile protein expression, e.g., the elevation of β-MHC protein, PGC-1α overexpression regulates different programs that are involved in contractile function of the heart.

Finally, several of our findings support that the age-associated cardiac remodeling differs between the MTg and the other two lines. In contrast to the old WT and MKO mice, which both show the same features typical of pathological cardiac hypertrophy, possibly associated with their higher diastolic blood pressures, the results obtained for the old PGC-1α-overexpressing mice suggest a more physiological form of hypertrophy (Ellison et al., 2012). First of all, physiological hypertrophy is characterized by improvements in oxidative phosphorylation, calcium handling as well as reduced fibrosis and apoptosis (Mann and Rosenzweig, 2012). Several of these traits were indeed found in our MTg line. Second, increased mTOR expression has been linked to physiological eccentric hypertrophy (Gielen et al., 2010; Ikeda et al., 2015) and was also observed in our MTg mice. Furthermore, the increase in VEGF expression in the MTg mice could be indicative of a higher tissue vascularization, another feature of physiological cardiac hypertrophy. Consistent with our findings, restoration of cardiac levels of PGC-1α resulted in an increase in VEGF and a preservation of capillary density in mice with a transverse aortic constriction (Pereira et al., 2014). Finally, the changes in calcium handling proteins observed in the MTg animals suggest improved E-C coupling, in line with observations done in exercise-induced physiological hypertrophy of the heart (Mutikainen et al., 2016).

Collectively, the results obtained in the MTg mice suggest that modest overexpression of PGC-1α in the heart is sufficient to prevent many adverse changes evoked by aging in the heart while others remain unaffected, or even show an unexpected phenotype (e.g., heart rate). Of note, some of the potentially beneficial effects are already observed in young MTg animals, and are preserved into old age. Inversely however, the mild reduction in PGC-1α transcript levels in the MKO mice elicited a more restricted response, even though this reduction of ~40–50% quantitatively recapitulates the decrease in PGC-1α gene expression in different contexts and models of cardiac pathologies (Garnier et al., 2009; Schilling et al., 2011). The requirement for adequate PGC-1α gene expression was most notable in the regulation of several mitochondrial genes. Most of the other parameters that were studied here were unaffected by the reduction in PGC-1α in the MKO model, with the exception of TERF1. This surprisingly mild phenotype could be caused by limitations of the animal model, which harbors a life-long reduction in PGC-1α that could trigger adaptive responses, or in which the reduction might simply be insufficient to induce a more severe aging phenotype in the heart. Unfortunately, cardiac-specific knockout mice for PGC-1α have not been reported yet, and therefore, the phenotype of mice with a complete ablation of PGC-1α in the heart is unknown. Second, the cardiac phenotype of the MKO and the MTg animals might be confounded by the more marked knockout and overexpression in skeletal muscle, e.g., by modulation of the secretion of myokines or differences in locomotion and endurance. Furthermore, it is conceivable that the numerous compensatory, redundant, adaptive, maladaptive and reparative mechanisms, which are engaged to provide cardio-protection in a pathological context (Strait and Lakatta, 2012), circumvent an absolute requirement for PGC-1α to ensure proper regulation of different biological programs. In line with this hypothesis, whole body ablation of PGC-1α results only in a mild phenotype, which is dramatically exacerbated in a PGC-1α/PGC-1β double-knockout context (Lai et al., 2008). Then, aging is a progressive phenomenon, in which most aging-linked changes are initiated and escalated at the age of 1–24 months in C57Bl6/J mice (Flurkey et al., 2007). Based on the time of sacrifice, we obviously can only draw direct conclusions from our data on hearts of mice at the age of 24 months. It is entirely possible that these effects might differ in quantitative and qualitative outcome at earlier or later time points. Finally, it is obviously conceivable that cardiac PGC-1α is largely dispensable for the aging phenotype.

## Conclusion

PGC-1α is a pleiotropic transcriptional coregulator that controls complex networks and biological programs in various tissues (Kupr and Handschin, 2015). Using genetic mouse models to mimic the reduction in cardiac PGC-1α expression in pathological contexts and the increase in PGC-1α transcript levels in exercise, respectively, we have now delineated the contribution of this coactivator to the aging response of the heart. The MKO mice exhibited a precocious aging phenotype in regard to mitochondrial gene expression, but as a single hit, a reduction in cardiac PGC-1α is insufficient to trigger or exacerbate the adverse remodeling of older hearts. Strikingly however, the MTg animals were largely protected against the pathological plasticity induced by aging, indicating that elevation of PGC-1α, e.g., as observed after endurance training, could be a potential intervention to mitigate the decline in heart function and reduce aging-associated cardiac pathologies. Thus, since our analyses were largely focused on the transcriptional program, future studies should aim at a more functional assessment of contractile function, potentially combined with interventional approaches to boost cardiac PGC-1α levels.

## Author contributions

JG, NW, MB, and CH designed the study. NW performed the experiments. NW, JG, MB, and CH analyzed and interpreted the data. NW and CH wrote the manuscript.

### Conflict of interest statement

The authors declare that the research was conducted in the absence of any commercial or financial relationships that could be construed as a potential conflict of interest.

## Abbreviations

BP: blood pressure
E-C coupling: excitation-contraction coupling
ECM: extracellular matrix
FA: fatty acid
FAO: fatty acid oxidation
MTg: muscle-specific PGC-1α transgenic
MKO: muscle-specific PGC-1α knockout
OXPHOS: oxidative phosphorylation
PGC-1α: peroxisome proliferator-activated receptor γ coactivator 1α
SR: sarcoplasmic reticulum
VEGF: vascular endothelial growth factor
WT: Wild type.
